# Getting older can be exhausting

**DOI:** 10.1186/s13054-014-0465-5

**Published:** 2014-07-29

**Authors:** Rohit Mittal, Mandy L Ford, Craig M Coopersmith

**Affiliations:** Department of Surgery and Emory Critical Care Center, Emory University School of Medicine, 101 Woodruff Circle, WMB Suite 5105, Atlanta, GA 30345 USA

## Abstract

Sepsis is a disease that affects primarily the aged. Although mortality is higher in both older septic patients and aged septic mice, the mechanisms underlying decreased survival in older hosts are incompletely understood. New work by Inoue and colleagues demonstrates persistent inflammation and T-cell exhaustion in older septic patients and aged septic mice. The clinical significance of these findings is manifested not only in increased mortality but also in a marked difference in secondary infections in older patients as long as a month following ICU admission.

Sepsis predominantly afflicts patients at the extremes of age, both old and young. In the previous issue of *Critical Care*, Inoue and colleagues [1] explored the relationship between aging and sepsis by examining septic ICU patients and mice subjected to cecal ligation and puncture, to understand potential reasons why aging is associated with substantially worse outcomes. In the US, although older patients account for slightly more than 10% of the population, greater than 60% of cases of sepsis and nearly 80% of deaths occur in patients over 65 years of age [2,3]. The observation that aging is associated with mortality has been replicated in multiple murine studies of sepsis [4,5], and it has been suggested that using young mice as surrogates for older patients is one of many reasons why positive preclinical trials in sepsis have failed to translate into therapeutic benefit at the bedside [6]. At baseline, aging, in isolation, leads to activation of the innate immune system, a phenomenon termed ‘inflamm-aging’ [7]. This inflammation is further upregulated following sepsis in mice [8], although its significance in mediating mortality is unclear [9]. Notably, aging is also associated with marked proteomic differences as well as decreased numbers of immunocompetent T cells in patients with sepsis [10,11].

To expand our current knowledge, Inoue and colleagues [1] examined septic patients (greater than or less than 65 years of age) and healthy age-matched controls and performed further mechanistic studies in aged and young septic mice. The increase in mortality seen in both older patients and aged (20- to 22-month old) mice correlated with increases in the inflammatory cytokines IL-6, monocyte chemotactic protein 1, and IL-10, with differences occurring as soon as 6 hours and persisting for 6 days. However, increasing evidence also suggests that sepsis induces a state of immune suppression with impaired immune cell effector function [12] and that this can coexist with a state of persistent inflammation [13]. Consistent with this, the authors demonstrated that aging and sepsis not only induced persistent hyperinflammatory changes but also resulted in a hypoinflammatory immunosuppressive state. Specifically, both older septic patients and aged septic mice exhibited increased expression of the co-inhibitory receptors programmed cell death protein 1 (PD-1) and cytotoxic T-lymphocyte antigen 4 on peripheral CD4^+^ T cells. Along with this increased co-inhibitory receptor expression, CD4^+^ T cells had impaired IL-2 production and reduced proliferation. Because upregulation of the co-inhibitory receptors PD-1 and B- and T-lymphocyte attenuator has been shown to be associated with T-cell exhaustion and contribute to sepsis morality [14,15], the finding that these are differentially regulated in aging may have a high degree of clinical significance. Supporting this contention is the authors’ observation that older patients exhibited a marked increase in the incidence of secondary infections. Two weeks after identification of sepsis, 82% of older patients had a secondary infection compared with only 21% of patients less than 65 years of age. A large discrepancy in the incidence of secondary infections was also noted 1 month after ICU admission. These remarkable findings provide strong evidence for an ongoing immunosuppressive state in older septic patients that is not reversed with standard therapy in the ICU.

In their study, as with any novel study, a number of opportunities for future research remain. Recent work demonstrates the importance of effector memory CD8 T cells in sepsis [16], and because frequencies of effector memory CD8^+^ T cells increase with age, examining these cells in aging and sepsis would be of great interest. Additionally, work in models of chronic viral infections has revealed that T cells have the potential to express multiple co-inhibitory receptors that can have synergistic inhibitory effects [17]. Thus, assessing the constellation of co-inhibitory molecules expressed by aged T cells during sepsis may provide new clues to help explain the immunosuppressed state of older patients with sepsis. Finally, it should be noted that the number of patients examined in this single-center trial is relatively small and therefore the results should be viewed as preliminary, requiring validation across different centers. For now, this important bedside and bench study yields novel insights into how inflammation and immunosuppression are both augmented in aging and sepsis and yields potential pathways that might be exploited for therapeutic gain in older patients with sepsis.

## Abbreviations

IL: Interleukin
PD-1: Programmed cell death protein 1
